# Translational science at the undergraduate level: awakening talents to overcome the valley of death - case report

**DOI:** 10.1590/1678-9199-JVATITD-2025-0005

**Published:** 2025-05-23

**Authors:** Rui Seabra Ferreira, Cristina Kampus Mantovani, Ana Silvia Sartori Barraviera Seabra Ferreira, Laura de Oliveira Nascimento, Merari de Fátima Ramires Ferrari, Daniel Fabio Kawano, Katlin Brauer Massirer, Gabriel Forato Anhê, Rosley Anholon, Celso Pereira Caricati, Luciane Meneguin Ortega, Sarah Guilbert, Teresa Lambe, José Paes Oliveira-Filho, Sue Ann Costa Clemens, Benedito Barraviera

**Affiliations:** 1 Center for the Study of Venoms and Venomous Animals (CEVAP), São Paulo State University (UNESP), Botucatu, SP, Brazil.; 2 Botucatu Medical School, São Paulo State University (UNESP), Botucatu, SP, Brazil.; 3 State University of Campinas (UNICAMP), Campinas, SP, Brazil.; 4 University of São Paulo (USP), São Paulo, SP, Brazil.; 5 Department of Translational Medicine, School of Medical Sciences, State University of Campinas (UNICAMP), Campinas, SP, Brazil.; 6 Center for Molecular Biology and Genetic Engineering (CBMEG), Center for Medicinal Chemistry (CQMED), State University of Campinas (UNICAMP), Campinas, SP, Brazil.; 7 Nuffield Department of Medicine at Oxford University, United Kingdom.; 8 Department of Pediatrics and Oxford Vaccine Group at the University of Oxford, United Kingdom.; 9 Department of Veterinary Clinical Science, School of Veterinary Medicine and Animal Science (FMVZ), São Paulo State University (UNESP), Botucatu, SP, Brazil.; 10 The Oxford Research Group LATAM, University of Oxford, United Kingdom.

**Keywords:** Translational science, Valley of death, Undergraduate course, CDMO

## Abstract

**Background:**

In the biomedical field, translational science is the process of applying basic scientific knowledge to advance clinical research through the creation of new drugs, devices, medical procedures, preventive measures, and diagnostic kits. The Covid-19 pandemic exposed a shortage of professionals trained in translational research, essential for responding to global demands. To drive advancements, researchers must overcome the ‘valley of death’, a critical phase in clinical investigation. In response, CEVAP at São Paulo State University (UNESP), Botucatu, Brazil, has developed a strong 'knowledge industry' centered on Translational Science. As part of its research and innovation efforts, CEVAP has developed two biopharmaceuticals, the fibrin sealant and the apilic antivenom, which are currently in the final stage of development. In 2024, CEVAP began the first Brazilian Contract Development and Manufacturing Organization (CDMO) for developing and producing validated and qualified pilot-scale batches to generate clinical trial material.

**Case Presentation:**

The implementation of the optional undergraduate course in Translational Science marks a crucial step in strengthening the ‘knowledge industry’. The program, developed in collaboration with São Paulo’s three public universities (USP, UNESP, and UNICAMP), also involves an international partnership with the University of Oxford’s Department of Pediatrics and the Oxford Research Group LATAM. The successful launch of this course underscores its importance in interdisciplinary education and institutional collaboration. By bridging gaps between research and application, the program equips professionals to meet the growing demand for expertise in translational science. Given the project's success, it will transition into a one-year ‘Qualification in Translational Science’, available to students enrolled in São Paulo state universities.

**Conclusion:**

The preparation of these professionals will be strategic for transforming basic research into products for health, saving lives, and combating future pandemics that will emerge around the world.

## Background

Translational science, also known as translational research, aims to convert research results into products or processes that directly benefit animals and human beings. In biomedical sciences, this process is often referred to as moving ‘from bench to bedside’ [1, 2]. Although the term *‘*translational*’* is recent, Louis Pasteur stated that ‘there is neither basic nor applied science, but rather only applications of science’ [3]. The late 19^th^ century was rich in scientists, who, through practical examples, implemented the ‘application of science’. Notable figures from this period included Louis Pasteur, Robert Koch, Camille Guérin, Joseph Lister, Paul Ehrlich, Alexander Fleming, Albert Calmette, among others.

At the beginning of the 20^th^ century, many Brazilian scientists embraced the paradigm of applied science and left a unique legacy in the fight against tropical diseases, especially the endemic diseases that have and continue to affect our country. We can quickly list Vital Brazil, Oswaldo Cruz, Carlos Chagas, Adolfo Lutz, Rocha e Silva, Emílio Ribas, and more recently, Ciro Carlos Araújo de Quadros, known for his work in the global eradication of polio. We must boldly state that Vital Brazil was the main translational scientist in Brazilian history. In addition to discovering the specificity of antivenoms, he established the production platform for these immunobiologicals from horses - a method still used today, due to its robustness. This platform continues to deliver important immunobiologicals that are saving lives in the field [4, 5].

In the past century, due not only to the increase in discoveries of new medicines but also because of the need to carry out tests on human beings, the ‘valley of death’ of clinical research was born. This is due to the construction of ethical and regulatory principles throughout history, in addition to the challenges imposed by the need for high financial investments, the excessively long time for product development, the high failure rate, and finally the complex bureaucracy.

Ethical principles that emerged from 1900 onwards underwent a substantial increase in 1947 after World War II, when the Nuremberg Military Tribunal published the Nuremberg Code [6]. In 1948 the United Nations General Assembly drafted the Universal Declaration of Human Rights (UDHR) and in 1964 the General Assembly of the World Medical Association published the Declaration of Helsinki, which established the ‘Ethical Principles for Medical Research on Human Beings’ [7]. Essentially, these guidelines were based on a tripod: approval of the project by peers, consent of research subjects, and confidentiality of the individual data obtained. 

These regulatory principles date back to 1906, when the U.S. President Theodore Roosevelt signed the Wiley Act, granting administrative, regulatory, and supervisory powers to the newly formed Bureau of Chemistry. In 1930 this Bureau was renamed The Food and Drug Administration (FDA). In 1962, the FDA set forth minimum guidelines for conducting clinical trials aimed at registering medicines. From then on, any medicine seeking FDA approval must undergo rigorous tests to demonstrate safety, quality, and efficacy [8]. 

In Brazil, the ethical principles were consolidated in 1996, when the National Health Council established guidelines and standards for research involving human subjects’ beings. During this period the National Research Ethics Council (CONEP) was created, linked to the National Health Council through CNS resolution no. 196/1996 [9].

Regulatory principles were established in 1999 with the creation of the National Health Surveillance Agency (ANVISA) through law no. 9,782 of January 26, 1999 [10]. The agency gained international respectability and in 2012 proposed a coalition to deepen cooperation among medicinal regulatory authorities during the 65^th^ World Health Assembly. The coalition was created in December 2013 by eight regulatory authorities named The International Coalition of Medicines Regulatory Authorities (ICMRA). The year 2023 marked the 10^th^ anniversary of the entity, whose mission is to ‘respond to the needs of a system of global governance and more effective cooperation strategies’. Currently made up of 38 participants and is chaired by the European Medicines Agency (EMA), with the co-chairmanship of ANVISA and the Pharmaceutical Products and Medical Devices Agency (PMDA) of Japan. Given its history of success, ANVISA has achieved the position of ‘strict regulatory authority’ (SRA). The World Health Organization (WHO) classifies a strict regulatory authority as ‘a national medicines regulatory authority that applies rigorous standards of quality, safety, and usefulness in its regulatory assessment of medicines and vaccines for market approval’. 

## Bridging the valley of death

The ‘valley of death’ refers to the challenges researchers face in transferring the discovery of a candidate molecule from the laboratory bench through development, pre-clinical, and clinical trials, to finally registering and making the product available to the population. The term emerged around 1990 [11], gained prominence in 2008 [12], and peaked at the end of the last decade [13-15]. During the pandemic, it gained further notoriety, highlighting the urgent need to invest in education and training to build a diverse and highly qualified translational scientific workforce to overcome these challenges [16-20].

According to Sun et al. [21], ‘Ninety percent of clinical drug development fails despite implementation of many successful strategies, which raises the question as to whether certain aspects in target validation and drug optimization are overlooked’. This became evident in the early 2000s when Batta et al. [22] verified the approval by the Center for Drug Evaluation and Research (CDER) - a division of the Food and Drug Administration (FDA) - of just 511 drugs between 2000 and 2017. Between 2000 and 2008, 209 were approved, of which 9.09% were for cardiovascular diseases, 12.91% for neurological diseases, 5.26% antibiotics, 5.74% antivirals, 11.96% anticancer drugs and 7.17% biological medicines. Between 2009 and 2017, 302 medicines were approved: 5.29% for cardiovascular diseases, 9.93% for neurological diseases, 5.29% antibiotics, 5.96% antivirals, 17.54% anticancer drugs and 15.56% biological medicines. Between 2018 and 2022, which included the time of the pandemic, the CDER approved 247 new medicines [23]. These results show an upward curve in the approval of new medicines, beginning with an approval rate of 23.2 medicines/year between 2000 and 2008, followed by 34.5 medicines/year between 2009 and 2017, and 49.5 medicines/year between 2018 and 2022. The data also reflects increased investments in research and development for products against cancer and for biological medicines.

Since the concept of ‘valley of death’ was proposed in the early 1990s [11], most authors [12-19] have described this challenging phase as the gap between basic and applied research. However, in 2015 Kimmelman and London [24] proposed a new ‘spectrum’ for translational research, identifying at least four stages, or mini ‘valleys of death’, labeled T1, T2, T3, and T4:

T1: translation to healthy humans (from discovery to phase I clinical trial),
T2: translation from healthy to sick individuals (phase II and III clinical trials),
T3: translation from patients to daily practice (phase IV clinical trial and their application),
T4: translation to the healthy population (from phase IV to studies in the healthy population).

**Figure 1.**
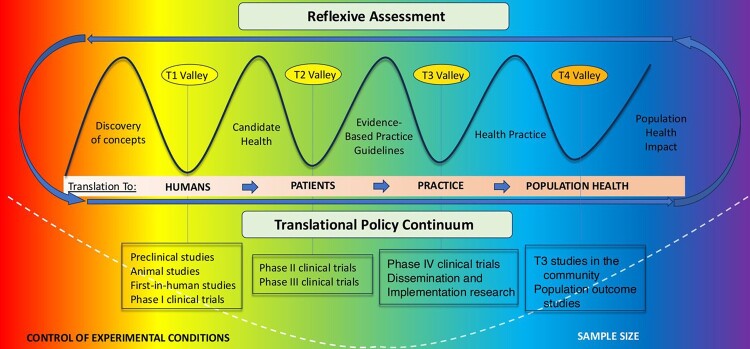
Translational valleys (T valley) on translational research spectrum, adapted from the University of California [25].

According to Kimmelman and London [24] “Scientists describe this multi-phase process as the ´translational spectrum´ or ´translational pipeline´”. Each metaphor highlights a different aspect of the process but, either way, the goal is to move scientific discoveries ‘from bench to bedside’ - which is to say, from the laboratory or academic setting into the actual healthcare field - as quickly and safely as possible. In other words, basic research remains a fundamental part of this process, making universities crucial players in this scenario. Thus, Translational Science is a continuum process with some aspects in the Translational Valleys (T Valley) identifiable on the Translational Research Spectrum (Figure 1) [25].

Translational science must navigate the ‘valley of death’ to ensure that products or processes are competitive and reach the consumer. In this continuous process of Translational Science, and in the challenges to bring innovations to the final consumer, universities play a crucial role: acting as catalysts for culture and entrepreneurial practices and helping researchers transform science and technology into innovations that benefit society.

This worrisome scenario was exacerbated by the Covid-19 pandemic officially declared by the WHO in March of 2020 [26]. On that occasion, humanity suddenly found itself facing a deadly enemy that required new protective equipment, to test the repositioning of drugs, to standardize new diagnostic techniques, and finally to develop safe and effective vaccines and medicines - all in an emergency context. And this was accomplished thanks to the effort and creativity of scientists, universities, pharmaceutical industries, regulatory agencies, and governments that organized and came together to accelerate the development process. The academic and corporate worlds were faced with a lack of professionals and infrastructure capable of taking on this challenge. Dozens of suggestions for training in translational science were proposed [27-29] including even changes in researchers' evaluation metrics [30]. 

To overcome this health emergency, the speed used to resolve the various problems presented was unimaginable. One of them already existed but had not yet been recognized to receive the notoriety it deserved. They were the unknown CDMOs (Contract Development and Manufacturing Organizations) [31]. Some of them had been created since 2010 and their mission was to offer outsourcing services to large pharmaceutical and biotechnology companies. The basic objective was to accelerate the development of medicines and shorten the time spent between the laboratory bench and the treatment and prevention of disease [31]. 

In this scenario, São Paulo State University (UNESP), in Brazil, had a very positive and outstanding role. In 2022, the Center for the Study of Venoms and Venomous Animals (CEVAP) - a research and innovation institute at UNESP based on the Botucatu Campus, São Paulo, Brazil - began construction of the first Contract Development and Manufacturing Organization (CDMO) in Latin America, whose building was completed on June 13, 2024 [32]. Its mission will be to: establish a bridge between basic and applied research; produce clinical trial material validated for phase I, II and III clinical trials; encourage clinical trials of genuinely Brazilian products; stimulate the development of drugs aimed at treating neglected tropical diseases; encourage graduate programs to carry out validated clinical trials; train qualified professionals to face the challenge of the ‘valley of death’; stimulate research, development and innovation at the national level; alleviate the Brazilian trade balance deficit, and finally generate wealth for the country by attracting national and international investments and establishing public-private partnerships [32]. To complete this rich ecosystem, support from development agencies was needed. 

In 2021, the São Paulo Research Foundation (FAPESP) approved the Center for Translational Science and Development of Biopharmaceuticals (CTS-CEVAP). This center - which includes researchers from the Federal University of São Paulo, University of São Paulo, and from three other institutions namely Biological Institute, Adolfo Lutz Institute, and Emílio Ribas Infectious Disease Institute - will be supported by FAPESP’s Science Center for Development Program, and based at the CEVAP at UNESP, Botucatu, SP, Brazil. The mission of CTS-CEVAP will be to produce clinical trial material for biopharmaceuticals and vaccine candidates. The goal will be to help researchers and startups bridge the ‘valley of death’ in clinical trials [33].

In 2022, the Coordination for the Improvement of Higher Education Personnel (CAPES) approved scholarships for students in the professional graduate program in clinical research at UNESP, valid for the next five years. This achievement will help students develop their projects on the ‘factory floor’ of the first Brazilian CDMO [32], inaugurated in 2024.

## Case presentation

To complete this ‘knowledge industry’, it was also necessary to innovate and create experimental programs for undergraduate students. Generally, undergraduate education does not emphasize independent research ability, innovative thinking and interdisciplinary cooperation and students are not prepared or encouraged to be part or coordinate innovative enterprises. This is further hindered by the lack of innovative culture in most universities in Brazil. 

Therefore, after strategic planning, in 2024 we created an innovative partnership between the three public universities in the state of São Paulo: the University of São Paulo (USP), the University of Campinas (UNICAMP), and the São Paulo State University (UNESP). Internationally, professors from the Department of Pediatrics at the University of Oxford and the Oxford Research Group LATAM were invited to participate. 

In March 2024, these universities began offering an integrated optional discipline for undergraduate students called Translational Science aimed at students in the areas of health, biological, agricultural, exact sciences, and engineering. The discipline is objectively aimed to awaken talent and provide translational professionals with specific training in translational science focused on pharmaceutical medicine and engineering applied to health. Therefore, it was offered to 30 students from the three Universities, consisting of synchronous online classes and three face-to-face visits to research laboratories at each of the Universities.

During the course, students were exposed to the theoretical and practical approach to research and development of pharmaceutical products and processes used in the diagnosis and treatment of diseases that affect human beings and animals. It covered topics from conception, development, pre-clinical and clinical stages, production, regulatory, and finally the registration of the new product or process.

Furthermore, it aimed to equip participants with fundamental skills and knowledge needed to drive discovery and encourage the development of new medicines. At the end of the discipline, the student should know not only the principles of prospecting, identifying and characterizing candidate molecules, but also good laboratory and clinical manufacturing practices, pre-clinical trials, production chains, ethical and regulatory principles involved, the challenges to overcome bureaucracy and ‘cross the valley of death of translational science’, and finally learn how to prepare protocols aimed at carrying out clinical trials from phase I to IV. This basic knowledge acquired will be the first step and serve as the framework of this long and arduous journey of translational science, that is, from the bench to the patient.

On March 1, 2024, the optional discipline began, registering 241 candidates for 30 vacancies offered - more specifically 8.03 students applying per vacancy. The content was divided into synchronous remote classes taught by professors from the three São Paulo state universities. The classes covered: Introduction to translational science (UNESP); Introduction to medications (UNICAMP); Pre-clinical development (Oxford); Introduction to drug preformulation (UNICAMP); ‘Valley of Death’ in clinical research (UNESP); Clinical development (Oxford); Manufacturing and quality control of vaccines, including topics on the development of vaccines against Covid-19 (Oxford); Basic concepts of entrepreneurship and technological innovation (USP) and Legal aspects on patenting, technology transfer and partnerships (USP).

Finally, the students participated in three in-person visits to USP, UNICAMP, and UNESP (Additional file 1). We believe that the main objectives of awakening talents and assessing students’ interest in the topic were achieved. The next step will be to develop a ‘Qualification in Translational Science’ that should last at least one year and will be offered to students who have already enrolled at USP, UNESP, and UNICAMP.

## Discussion

Throughout its 30 years of existence, CEVAP has built a robust ‘knowledge industry’ focused on translational science, offering specialization in diagnostic and therapeutic innovations (*lato sensu*) [34], and *stricto sensu* graduate courses - master's and doctorate degrees - within the context of clinical research [35]. As part of its research and innovation efforts, CEVAP has developed two biopharmaceuticals, the fibrin sealant [36] and the apilic antivenom [37], respectively. Both bioproducts are currently in the final stages of development. In 2024, CEVAP launched the first Brazilian CDMO [32] to develop and produce validated clinical trial material for products originating from Brazilian biodiversity, while also being able to meet demands from universities and pharmaceutical companies.

The establishment of the Translational Science discipline in 2024 represents a significant advancement in interdisciplinary education and inter-institutional collaboration. This pioneering initiative, a joint undertaking of USP, UNICAMP, and UNESP, in conjunction with international experts from the University of Oxford, seeks to address the escalating demand for professionals trained in translational science.

This case study underscores the efficacy of innovative pedagogical approaches in bridging the divide between theoretical knowledge and practical application within the fields of pharmaceutical medicine and health-related engineering. The comprehensive curriculum, encompassing molecular screening and preclinical trials through regulatory practices and clinical protocol preparation, fostered the development of both technical proficiency and essential soft skills in the students.

The program's notable success in attracting a high volume of applications per available place underscores the increasing recognition among students of the critical role of translational science in contemporary healthcare and biotechnology. The program's structure, which combined synchronous online instruction with practical laboratory sessions for a limited cohort of 30 students, facilitated the integration of theoretical knowledge and experiential learning - a crucial component in these application-oriented fields.

A key strength of this initiative is its focus on interdisciplinary learning, which facilitates the exchange of diverse perspectives among students from varied academic backgrounds, thereby promoting innovation. The collaboration with the University of Oxford, particularly in the instruction pertaining to Covid-19 vaccine development, provided students with invaluable real-world insights into one of the most significant health challenges of our time.

## Conclusions

The establishment of this optional undergraduate course in Translational Science marks a significant advancement in the development of a comprehensive biopharmaceutical ‘knowledge industry’ ecosystem. The high demand and subsequent performance of students from the three São Paulo state universities (USP, UNICAMP, and UNESP) suggest that the program has successfully engaged and trained motivated individuals in contemporary translational science. Moving forward, we aim to expand this initiative into a year-long ‘Qualification in Translational Science’ for students enrolled at our partner institutions. The Covid-19 pandemic highlighted the critical shortage of professionals equipped to conduct translational research and respond effectively to global health emergencies. The training of such professionals is therefore of paramount importance for mitigating the impact of future endemic or pandemic outbreaks.

## Availability of data and materials

 All data generated or analyzed during this study are included in this article.

## Supplementary material

The following online material is available for this article:

Additional file 1. Photographs of students participating in three in-person visits to USP, UNICAMP, and UNESP.
