# Supplementary material for: Translational science at the undergraduate level: awakening talents to overcome the valley of death - case report
Source: J Venom Anim Toxins Incl Trop Dis. 2025 May 23;31:e20250005. doi: 10.1590/1678-9199-JVATITD-2025-0005 (PMC12105585; doi:10.1590/1678-9199-JVATITD-2025-0005)
Supplement: Additional file 1. [file 1678-9199-jvatitd-31-e20250005-s1.pdf]

J Venom Anim Toxins incl Trop Dis
https://doi.org/10.1590/1678-9199-JVATITD-2025-0005

Supplementary Material to "Translational science at the undergraduate level: awakening talents to overcome the valley of death – case report"

Additional file 1. Photographs of students participating in three in-person visits to USP, UNICAMP, and UNESP.

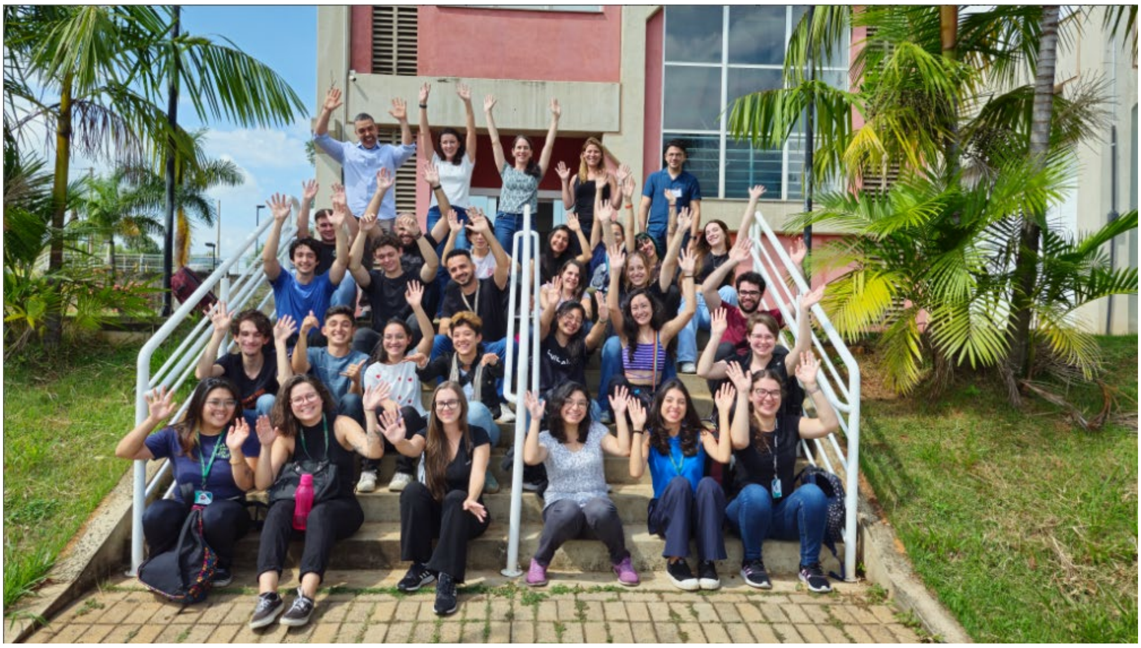

Figure 1. Students during a visit to UNICAMP.

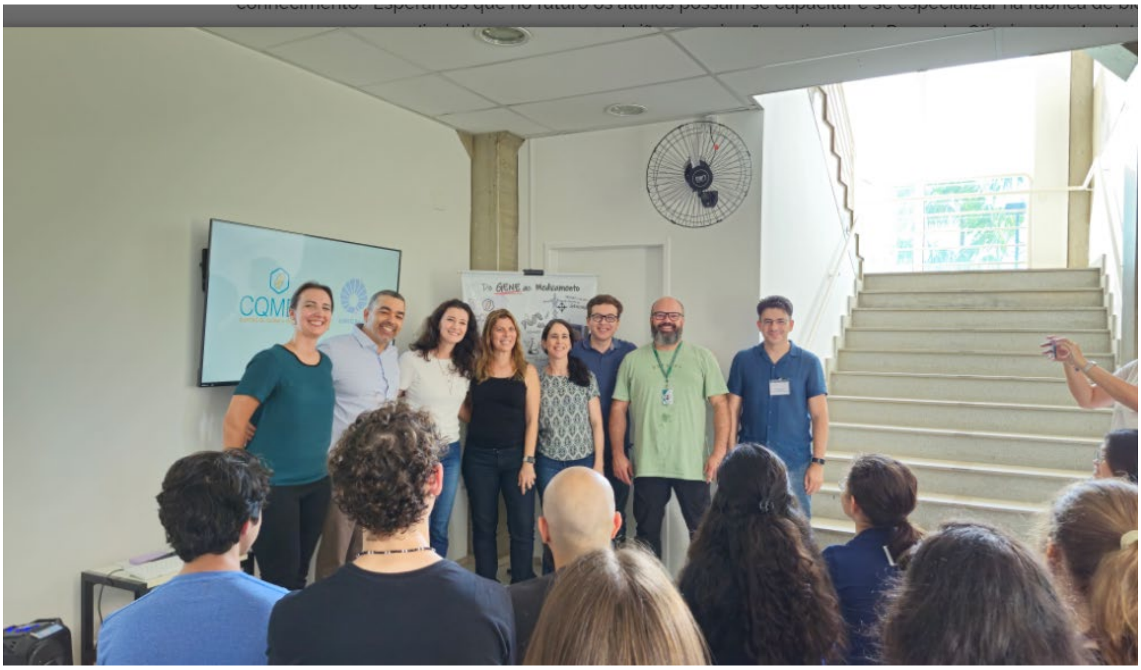

Figure 2. Professors during a visit to UNICAMP.

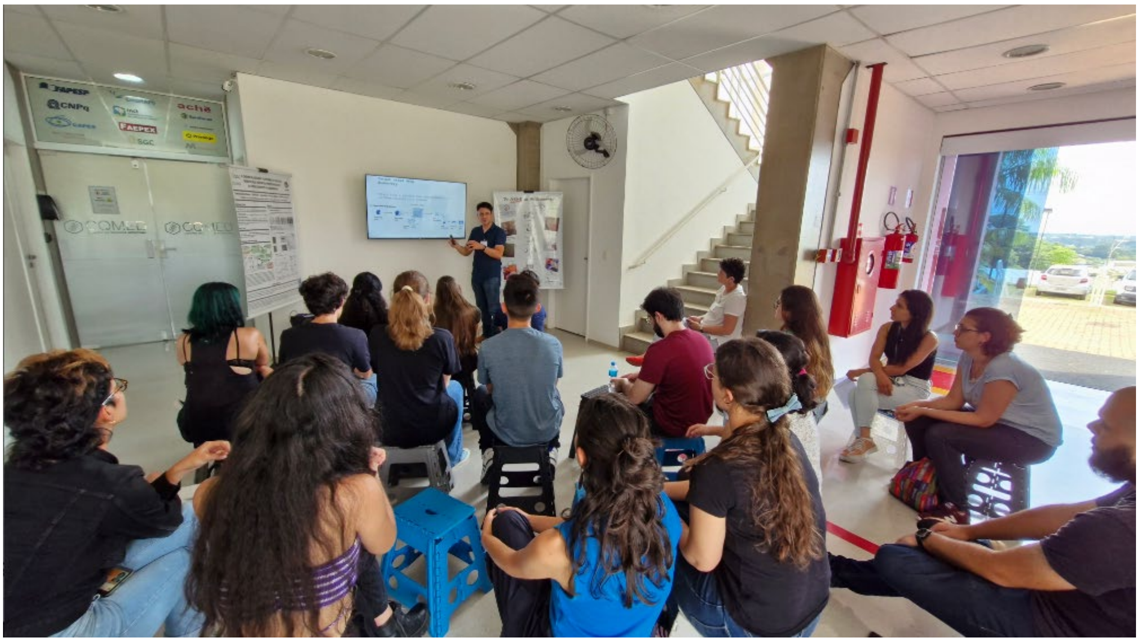

Figure 3. Students during a visit to UNICAMP.

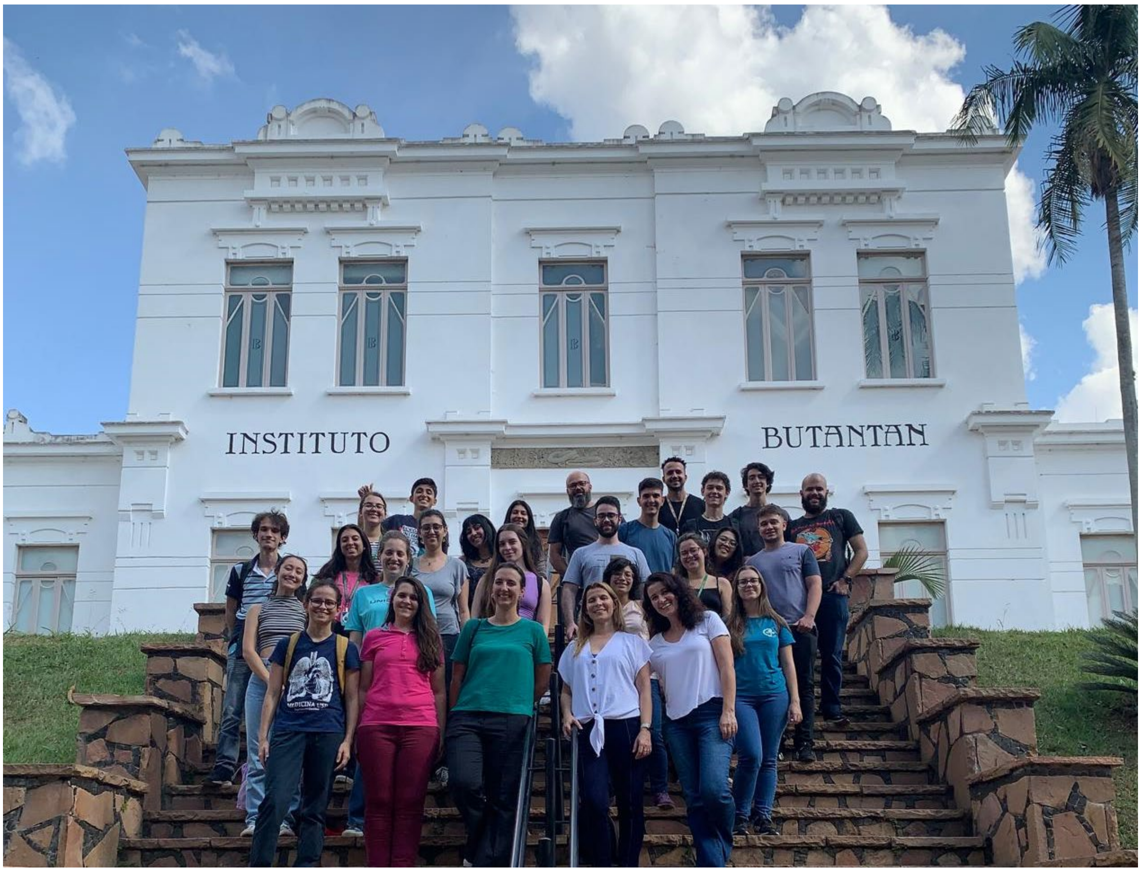

Figure 4. Students during a visit to Butantan Institute.

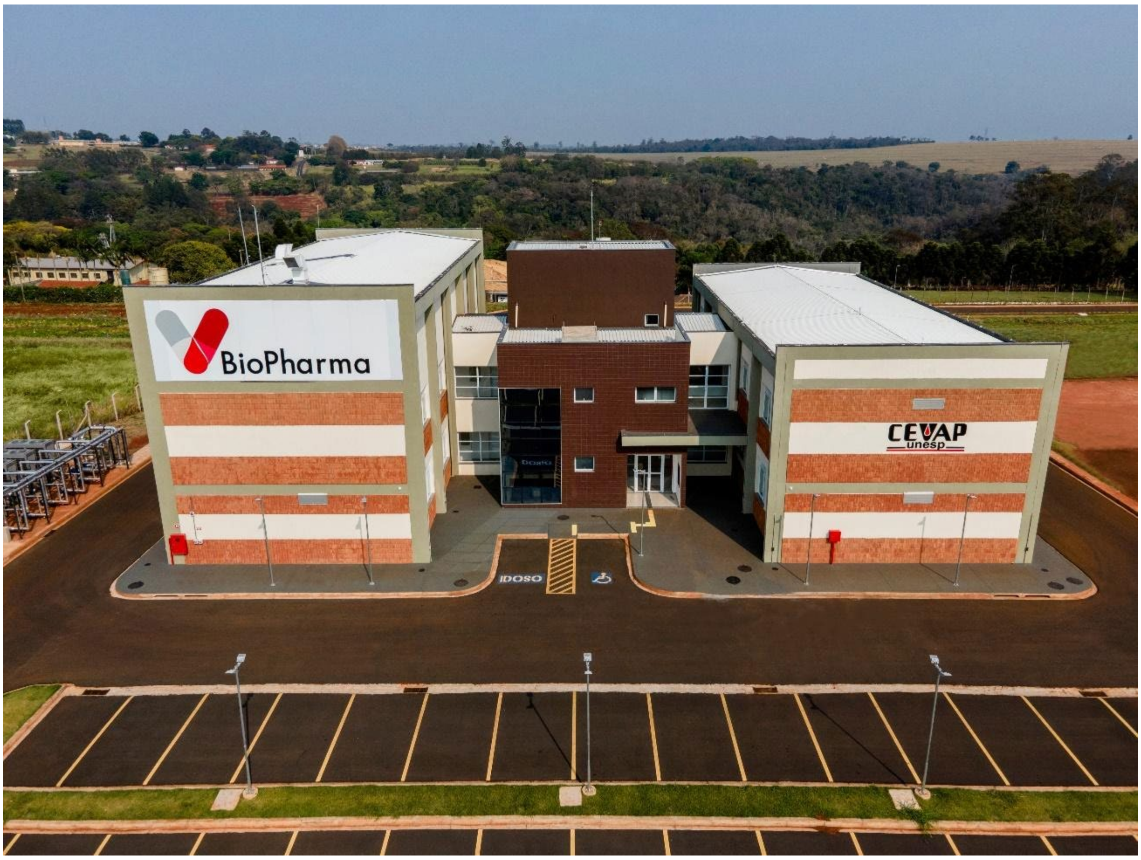

Figure 5. CDMO from CEVAP.

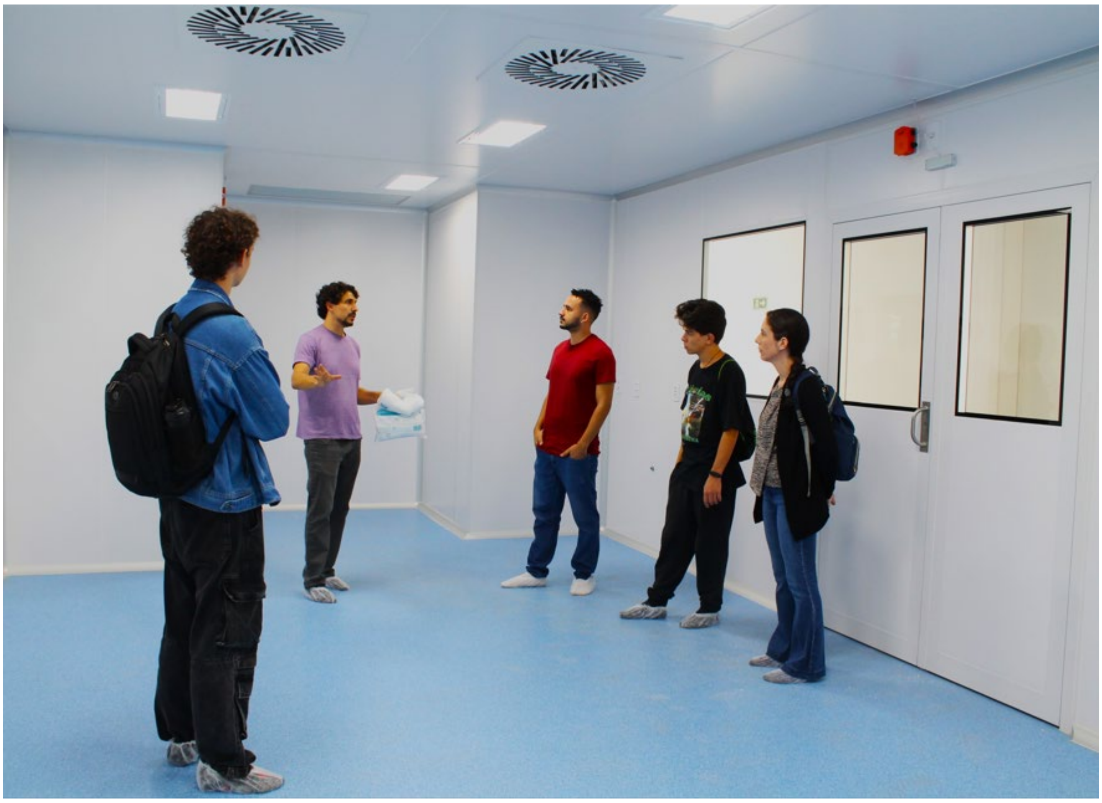

Figure 6. Students visiting the CDMO from CEVAP.

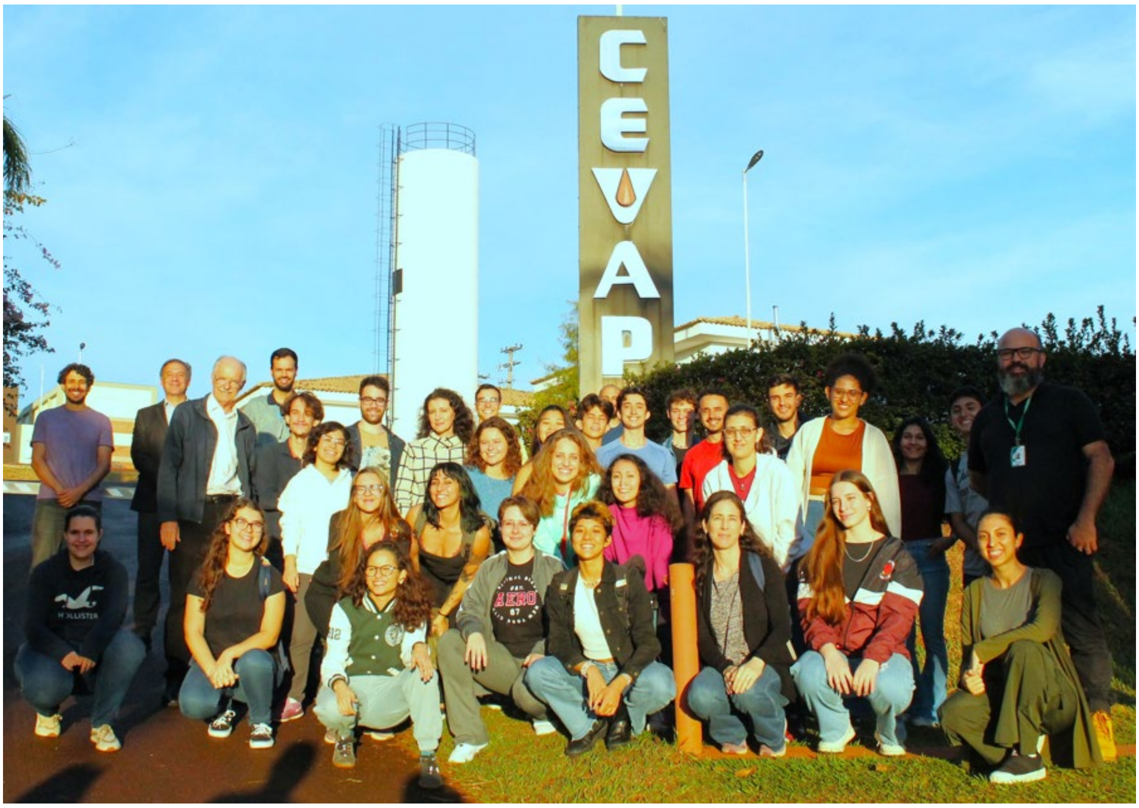

Figure 7. Students visiting CEVAP.
